# Deeper Insight into the Volatile Profile of *Rosa willmottiae* with Headspace Solid-Phase Microextraction and GC–MS Analysis

**DOI:** 10.3390/molecules27041240

**Published:** 2022-02-12

**Authors:** Ruifang Jiao, Ping Gao, Xinfen Gao

**Affiliations:** 1 Key Laboratory of Bio-Resources and Eco-Environment of the Ministry of Education, College of Life Sciences, Sichuan University, Chengdu 610065, China; 2019322040062@stu.scu.edu.cn; 2 CAS Key Laboratory of Mountain Ecological Restoration and Bioresource Utilization, Chengdu Institute of Biology, Chinese Academy of Sciences, Chengdu 610041, China

**Keywords:** *Rosa willmottiae*, volatile profile, flower development, SPME–GCMS

## Abstract

As the distribution center of *Rosa* in the world, China has abundant wild germplasm resources, which can contribute to the breeding of modern roses. To explore the potential value of wild roses distributed in the Sichuan–Tibet region, solid phase microextraction (SPME) and gas chromatography–mass spectrometry (GC–MS) were used to determine the volatile organic compounds (VOCs) in *Rosa willmottiae* flowers at three flowering stages (bud stage, initial flowering stage, full flowering stage). Meanwhile, we compared the VOCs of *R. willmottiae* with different phenotypes (double flowers and single flowers). A total of 74 volatile compounds were identified. The results show that the essential substances belong to alcohols and terpenoids. The main volatile organic compounds are 2-phenyl ethanol (20.49%), benzyl alcohol (10.69%), β-maaliene (8.66%), geranyl acetate (8.47%), and (+)-α-long pinene (6.127%). Different flowering stages had great influence on the volatile profile, from the bud stage to full flowering stage; the content of terpenoids released decreased by 6.17%, whereas alcohols and esters increased by 8.58% and 11.56%, respectively. The chemical diversity and the content of the main components with a different phenotype were not significantly different. Our result will provide a theoretical basis for the development and utilization of *Rosa willmottiae* in Sichuan and Tibet.

## 1. Introduction

Fossil finds prove that the *Rosa* species existed on earth at least 40 million years ago [1]. Rose is a common name given to the thorny shrubs and climbing vines of the genus *Rosa* in the Rosaceae family, which include about 200 known species of wild roses, mainly distributed in the temperate and subtropical regions of the Northern Hemisphere [2]. *Rosa* has been known since ancient times for its beautiful flowers and pleasant scent. Today, it is not only famous for its ornamental and aesthetic value in gardens, but also for its applications as an essential oil resource in the perfume, chemical, and cosmetic industries [3,4,5]. Nowadays, there are thousands of cultivated *Rosa* species through cultivation and propagation techniques [6]. China is the distribution center of *Rosa* in the world [7], rich in *Rosa* varieties (approximately 90) which have unique characteristics, and plays an important role in the modern rose cultivation history. Ten of the 15 important parents of modern roses come from China [8,9], which provided favorable conditions for cultivating garden roses [10,11] containing recurrent flowering, high central flower shape, and tea aroma characteristics and other excellent varieties. The first hybrid tea rose in the world, named ‘La France’, has a typical tea flavor which comes from one of the important parents, a Chinese *R. odorata* (‘Parks’ Yellow Tea-scented China’) [7,12].

*Rosa willmottiae* belongs to the subgenus Flora of the Rosaceae family, mainly distributed in Sichuan, Shaanxi, Gansu, Qinghai, and Tibet [2] and grows in shrubs at an altitude of 1300–3800 m. The type specimens of *R. willmottiae* were collected from Songpan County, Sichuan Province. According to records, *R. willmottiae* is a medicinal plant which can be used to treat feverish vomiting blood, hot polydipsia, epigastric distension, thirst, diarrhea, malaria, bleeding from knife wounds, and irregular menstruation [13]. Similar to other scented roses, *R. willmottiae* has a pleasant scent. However, its floral composition is rarely reported, only its petal aroma volatile organic compounds (VOCs) is [14], but how the volatile profile of the whole flower changes with the flowering stage is unknown. In addition, double flower mutants of *R. willmottiae* occur in the wild, but how the floral phenotype under natural mutation affects floral aroma components is also unknown.

Aroma volatile organic compounds are often difficult to measure, collect, and analyze, so they must rely on relevant technologies [7,15]. There are three methods for extracting floral components from plants: traditional water distillation, solvent extraction, and solid-phase microextraction (SPME) [16,17]. High temperature in traditional water distillation methods may result in the loss of some components, while solvent extraction methods often affect the determination of the plant VOCs due to residual organic solvents [18,19]. However, SPME can maximally maintain the integrity and authenticity of the flora VOCs [20]. This method is simple, rapid, and effective, with a low detection threshold [21].Therefore, SPME is the best method to extract volatile organic compounds and has been widely used [9,22]. Gas chromatography–mass spectrometry (GC–MS) is currently the most commonly used method for the determination of volatile substances [23].

In order to understand the flora volatile profile of *Rosa willmottiae*, this study used SPME + GC–MS to determine the main components: (1) to explore the changes of volatile organic compounds at different flowering stages [24]; (2) to analyze the effect of petal number variation on the fragrance composition of *R. willmottiae*; and (3) to excavate the floral components with economic value, to provide a scientific basis for developing new raw materials of plant essential oil, to cultivate new varieties, and to lay a theoretical foundation for the development and utilization of *R. willmottiae* in the Sichuan–Tibet region.

## 2. Results

### 2.1. Volatile Organic Compounds of Single-Petal Phenotype Rosebuds at Different Stages

**Figure 1:**
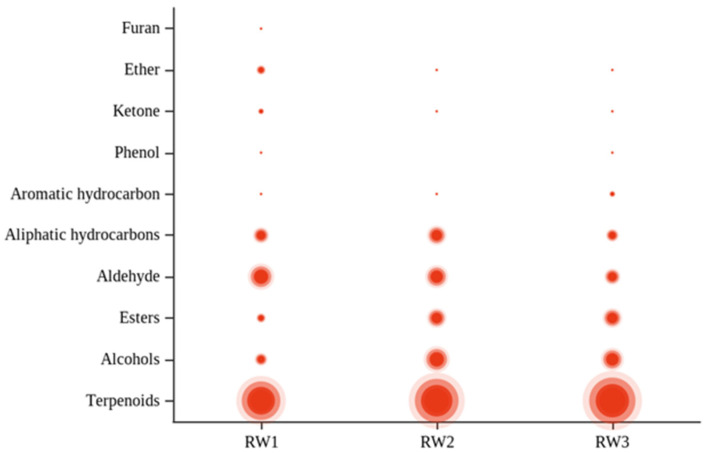
The substances’ richness of different kinds in *Rosa willmottiae* (RW1: bud stage, RW2: initial flowering stage, RW3: full flowering stage of single-flower phenotype).

**Figure 2:**
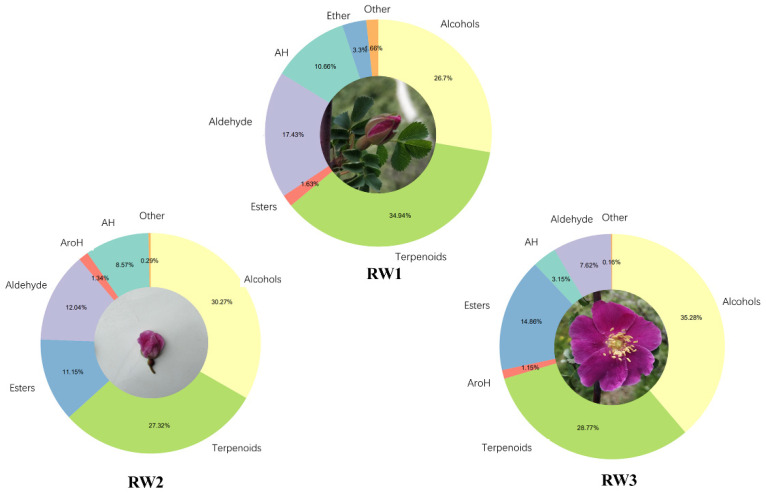
Ten kinds of VOCs’ accumulation in *Rosa willmottiae* at different stages.

A total of 74 volatile compounds were detected by using the SPME + GCMS (Table S1). These compounds belong to 10 categories, including alcohols, aldehydes, ketones, esters, ethers, phenols, terpenes, aliphatic hydrocarbons, aromatic hydrocarbons, and furans. Richness by category and total content at different flowering stages are shown in Table S2; RW1 to RW3 is composed of 8–10 categories (Figure 1) and the total richness of VOCs is (47 ± 2) species. Terpenes were the most abundant group in all stages (34.94% in RW1, 27.32% in RW2, and 28.77% in RW3), followed by alcohols (26.70% in RW1, 30.20% in RW2, and 35.28% in RW3) (Figure 2). Terpenoids were highest in RW1 and alcohols increased with flower blooming. The contents of aldehydes and aliphatic hydrocarbons decreased gradually with flower blooming. On the contrary, ester content gradually increased with flower blooming (3.3%, 11.15%, and 14.86%, respectively). The contents of others were present at low levels—less than 5% in total.

**Figure 3:**
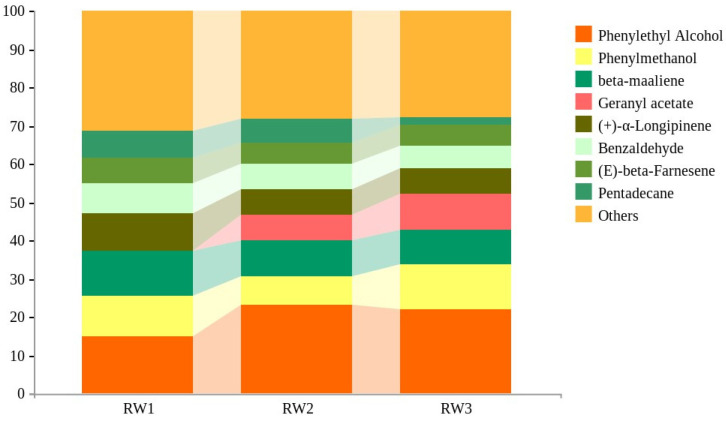
The relative content of main VOCs (>5%) in *Rosa willmottiae* at different stages.

The contents of the main chemical components of *Rosa willmottiae* at different flower stages are shown in Figure 3, and changes vary. Geranyl acetate (0%, 6.01%, 8.47%) gradually accumulates with flowers blooming; Although 2-phenylethanol has a similar trend, there is no significant difference between the initial stage and flowering stage (*p* > 0.05). On the contrary, the contents of β-maaliene (11.43%, 8.32%, 8.66%), (+)-α-longipinene (9.49%, 6.19%, 6.13%), (E)-β-farnesene (6.36%, 5.01%, 4.95%), and benzaldehyde (7.50%, 6.08%, 5.62%) decreased, also with no significant difference between the initial stage and flowering stage. Additionally, pentadecane (6.96%, 5.49%, 1.99%) decreased to an extreme extent with flower blooming. Benzyl alcohol (10.02%, 6.85%, 10.69%) content was the lowest in the initial stage.

**Figure 4:**
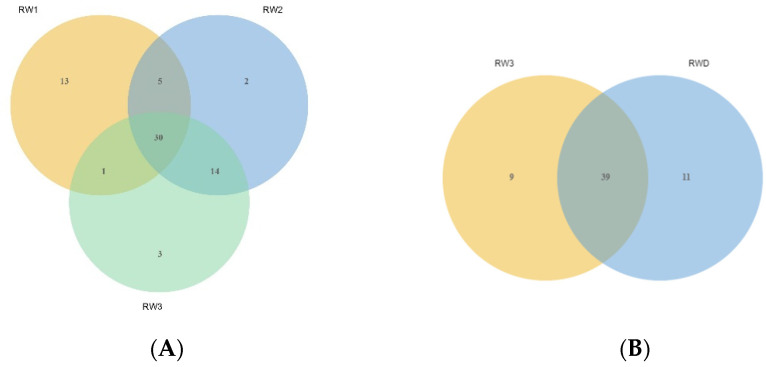
The number of substances in *Rosa willmottiae*. Venn diagrams (**A**) showing the numbers of common and unique substances at different stages. Venn diagrams (**B**) showing the numbers of common and unique substances at different flower phenotype.

There were 30 common components in the third stage of *Rosa willmottiae* (Figure 4A), including four alcohols (mainly 2-phenyl ethanol and benzyl alcohol), three kinds of aldehydes (mainly benzaldehyde and trans-2-hexenal), two aliphatic hydrocarbons (pentadecane and n-heptadecane), o-methyl benzene as the only common aromatic hydrocarbon, three esters (phenethyl acetate, benzyl acetate, and hexyl formate), one ether (anisole), and one ketone (2-methyl-4-undecanone). There were 15 common terpenes (mainly pinene and β-elemene). The unique components in bud stage were the most prevalent, up to 13 (β-bisabolene; α-muurolene; 1,3,5-trimethoxybenzene; methyl isoeugenol; tetradecane; trans-2,4-decadienal; 2-methyl-1-indanone; 1,3-cyclohexadiene, 5-butyl-; nonanal; (E)-2-octenal; (1S)- (1)-beta-pinene; 2-ethylfuran), and methyl isoeugenol (2.45%) had the highest content. The composition of alpha-gurjunene and isocaryophyllene is unique in the initial flowering stage, while 1, 2, 3, 4, 6, and 8 alpha-hexahydro-1-isopropyl-4, 7-dimethylnaphthalene, and β-humulene are unique to the full flowering stage. The initial and full flowering stages have common components, up to 85%.

### 2.2. Variation in Flowering Stage of R. willmottiae with Different Phenotypes

**Figure 5:**
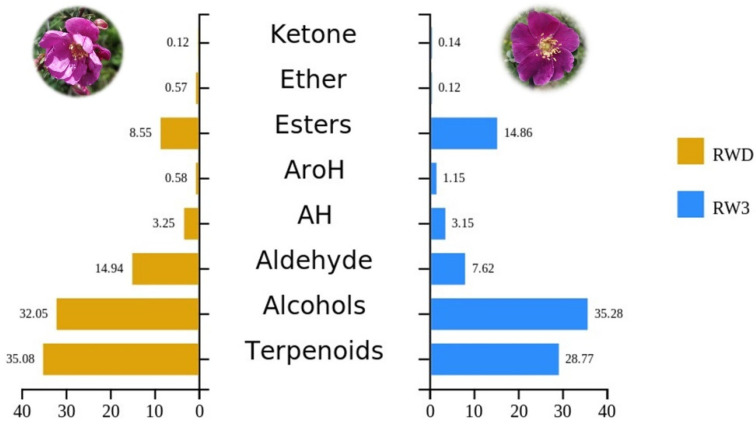
Comparison of different chemical classes’ content in single-flower phenotype and double-flower phenotype at full opening stage (AroH: aromatic hydrocarbons; AH: aliphatic hydrocarbons).

The single and double flower phenotypes of *Rosa willmottiae* contained roughly the same variety of volatile substances (Table S2). Terpenoids and alcohols were the most abundant. It can be seen from Figure 5 that, compared with RW3, the total contents of terpenoids and aldehydes in RWD increased by 6.31% and 7.32%, respectively, while the contents of alcohols and esters decreased by 3.23% and 6.31%, respectively. The contents of ketones, ethers, aliphatic, and aromatic hydrocarbons were all less than 5%. The alpha diversity analysis in Table 1 shows that the chemical components of the two phenotypic samples did not differ significantly. The Shannon index was 2.856 and 2.896, the Simpson index was 0.905 and 0.912, and the Pielou evenness index was 0.737 and 0.740, respectively.

There were 39 common components of the two phenotypic samples, accounting for more than 90% of the total components (Figure 4B; Table S1). Furthermore, phenethyl alcohol and benzyl alcohol were the main components, and there was little difference in the content. However, geranyl acetate was significantly higher in RW3 than RWD.

### 2.3. Principal Component Analysis (PCA)

**Figure 6:**
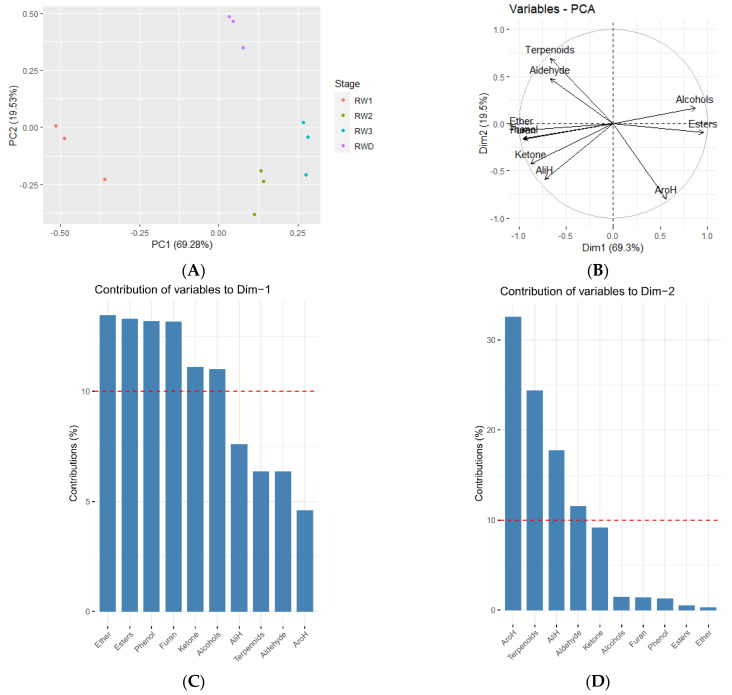
Principal component analysis (PCA) of *Rosa willmottiae* according to groups’ volatile compounds. (**A**): Score plot of *Rosa willmottiae*. (**B**): Biplot based on first and second components. (**C**,**D**): Percentages in brackets correspond to the explained variances of corresponding components or axes.

PCA analysis (Figure 6) of nine kinds volatiles at different stages and phenotypes in *Rosa willmottiae* could explain 88.81% of the total variation, with the ranking characteristics of the PC1 and PC2 first two restricted axes accounting for 69.3% and 19.5%. There was no overlap in principal component score plots for different stages and phenotypes, indicating difference in their volatile organic compounds and relative content compounds. Esters, alcohols, ethers, phenols, furans, and ketones had higher scores in PC1, which may help to distinguish the different blooming stage. The initial and blooming stage was positively correlated with alcohols and esters, and the bud stage was positively correlated with furans, phenols, ketones, ethers, and aliphatic hydrocarbons. The content of aromatic hydrocarbons, terpenes, fatty acids, and aldehydes was highest in PC2, which distinguished the different phenotypes. Among them, the double phenotype was positively correlated with terpenes and aldehydes.

## 3. Discussion

### 3.1. The Main Volatile Organic Compounds of Rosa willmottiae and Their Variation in Flowering Stages

The flora’s volatile profile varies in different plants and in different stages [25]; the difference not only depends on the plant’s physiological level, but is also concerned with their growth and reproduction strategies [26]. Most plants release volatiles gradually and generate aroma from the bud stage in order to obtain utmost pollination opportunities and reach the peak when the flowers fully open [25,27,28]. *R. willmottiae* has strong aroma in full bloom stage, with the accumulation of typical aroma components (benzyl alcohol, phenyl ethanol, ethyl acetate, etc.) in flowers, which is consistent with the pollination biological characteristics of the plant. The same phenomenon also existed in other plants [21,29,30,31,32]. Regulated by flower development, changes in plant growth hormone secretion, gene expression, and enzyme synthesis with time and environment at different flowering stages lead to variation in different metabolic pathways of composition, ultimately affecting the anabolism and release of volatile composition [33,34]. Ethylene can inhibit or promote the flower opening of rose species [35,36].The volatile organic compounds of *R. willmottiae* in bud stage were significantly different from those in initial and full flowering stages, which may be related to ethylene regulation. The specificity of enzymes showed that NADP/NAD involved in the mutual transformation of volatile alcohols’ and aldehydes’ [6] expression of OMT affects the production of 1, 3, and 5-trihydroxybenzene (a typical tea-scent aroma in Chinese tea rose) [17]. Therefore, we believe that the changes in the main components’ content of *R. willmottiae* are related to the level of some enzymes and genes, which can be further explored in the future.

Plant floral fragrances are characterized by high diversity; even if the flower type and color are similar, the flower fragrance is not exactly the same [37,38]. Some scent substances contribute to the unique fragrance of a plant; for example, 3,5-dimethoxybenzene and 1,3,5-trimethoxybenzene (phenolic, spicy, earthy, and animalic notes) endow the tea-scent perfume to the Chinese rose [9]. At present, more than 2000 fragrance substances have been identified [39], among which terpenoids are the largest class of substances [40]. In this study, apart from terpenes, *R. willmottiae* also has a high content of alcohols, with a total content of 58.15–66.16% (Figure 2; Table S2). Among them, the content of phenylethanol (sweet and rosy fragrance) is the highest in all stages and reaches its peak in full flowering stage. Additionally, benzyl alcohol (aromatic, floral, fruity), geraniol (rosy), β-maaliene (woody), (+)-α-long pinene (floral, rosy, fruity, raspberry), and acetic acid ethyl (fruity, rosy) constitute the main components of *Rosa willmottiae* and make the aroma strongest.

### 3.2. Heterogeneity of Volatile Organic Compounds in Different Phenotypes’ Flowers of Rosa willmottiae

In nature, the origin of double flowers mainly includes the accumulation, bracts, androgynies, stage, repeated, and inflorescence origins [41]. The study of hybrid rose petals confirmed that the double petals originated from stamen penalization [42]. The research on its formation mechanism mainly focuses on the ABCE model and proposes that the formation of the double flowers is caused by abnormal regulation of the ABCE gene during flower development [20,42,43], but the developmental model is incomplete. Factors such as leaf [44], temperature [45,46], and hormone [47] can directly/indirectly activate/inhibit the expression of genes related to floral organ formation, and thus regulate the formation and development of floral organs [41]. The ecological adaptations of plants and animals are usually observed in phenotypic traits and can be expressed by producing different biochemical substances [48]. There are few reports on double flowers from the chemical perspective. We assume that the polyphyll of wild rose flower influenced the VOCs. However, the heterogeneity analysis of volatile organic compounds showed that there was no significant difference in the total amount and main components of volatile organic compounds between the single petal phenotype and double petal phenotype; moreover, the chemical components’ diversity was basically the same. Therefore, the double flowers did not increase the content of volatile substances significantly, which was consistent with the study of *Rosa chinensis* [9]. They believed that double petals had a slower opening and release process than single petals, thus leading to the similarity. On the contrary, double-flower *Narcissus tazetta* released twice as many volatiles as single-flower *Narcissus tazetta* [49]. In summary, the relations between the petal duplication and flower volatiles should be taken into consideration. Although the double penalization of petals had no significant effect on the main volatile organic compounds content in *R. willmottiae*, further research can study this in depth through combination with biogeography, genetics, morphology, cytology, and other molecular mechanisms.

### 3.3. Potential Development Value of Floral Flavor Components in Rosa willmottiae

*Rosa* is an excellent horticultural plant in respect to its flower traits or fragrance and also its use as cut flowers. However, garden varieties lose fragrance due to breeders’ excessive focus on the flower shape rather than fragrance [6,50]. Fragrance is a sensory pleasure, one which can directly affect the part of the cerebrum that is in charge of memory and emotions, help people adjust psychologically, relieve tension, and benefit their immune regulation [51]. Therefore, *R. willmottiae* shows potential value for its bright, colorful petal and typical rose flavor. Apart from horticultural applications, it is well known that the *Rosa* oil possesses important economic value [52,53]. Essential oils are applied widely in cosmetics, pharmaceuticals, and food industries on account of their antibacterial, anticancer, neuroprotective, psychophysiological, and anti-aging activities [54], and *Rosa* oil is the most attractive, although it has low oil yield [7]. On account of the pleasant scent of *R. willmottiae*, we can attempt to explore its essential oil value as a raw material.

The results showed that 2-phenylethanol was the main component of *R. willmottiae.* Existing widely in the floral components of plants [55], 2-phenylethanol (2-PE) is an aromatic alcohol with a classical rose aroma [56,57]. It has been widely used as an aromatic alcohol in the food, pharmaceutical, and daily chemicals industries [58]. For example, in the food industry, it is used as an edible additive for blending the flavor of strawberry, caramel, honey, cream, etc. Its dosage has a clear national standard (GB 2760–2014) in China. In addition to traditional uses, its low volatility, high energy density, and non-hygroscopic properties have attracted interest in developing it as a potential biofuel molecule or a fuel additive [59]. Besides, 2-PE has good antibacterial properties [60], and researchers have confirmed through mouse experiments that phenylethanol has sedative effects [61]. Therefore, *R. willmottiae* can be used as a potential species resource to obtain natural phenylethanol.

With the exception of flower scent characteristics, *R. willmottiae* can grow well in southwest mountainous areas of China [2,62]. Therefore, *R. willmottiae* has the potential to provide a good hybrid source for cash crops of mountainous areas, and is of great significance to promote the economic development of southwest China.

## 4. Materials and Methods

### 4.1. Plant Materials

The *R. willmottiae* samples were collected from Aba Tibetan and Qiang Autonomous Prefecture, Songpan County, Sichuan Province in June 2020. The specimens were identified by Xinfen Gao, a senior researcher of the Chengdu Institute of Biology, Chinese Academy of Sciences, and preserved in the Herbarium of Institute of Biology, Chinese Academy of Sciences. In this study, flowers were divided into three stages [9,24] according to the degree of blooming: bud stage (RW1), the initial flowering stage (RW2), and full flowering stage, including the single flower phenotype (RW3) and double flower phenotype (RWD). The collected fresh flowers were wrapped in aluminum foil and immediately placed in liquid nitrogen. They were transported back to the laboratory and stored in −80 °C refrigerator as soon as possible.

### 4.2. Extraction and Analysis

In this study, SPME was used to extract VOCs from the samples we referred to [9], with minor modifications. Before extraction, the samples were ground into a fine powder with liquid nitrogen. Then, a 1.5–2 g sample powder was transferred to a 20 mL headspace and kept in a constant temperature water bath for balance at 50 °C for 20 min, then the extraction head (50/30 μm DVB/CAR/PDMS) was inserted and extraction conducted for 40 min. The solid-phase micro-extraction head was then inserted into the gas chromatographic inlet, and analysis was conducted at 270 °C for 2 min. Each stage had three replicates.

A GCMS-QP2010 system (Shimadzu, Japan) was used for compound identification. The GC–MS analysis conditions were as follows: The chromatographic column was a DB-5 quartz capillary column (30 m × 0.25 mm × 0.25 µm), the detector was a hydrogen flame ionization detector, high purity helium gas (99.999%) was used as a carrier gas with the gas velocity of 1.0 mL/min, and the split ratio was 3. The column temperature was held at 40 °C for 5 min, increased by 5 °C min^−1^ to 150 °C, increased by 10 °C min^−1^ to 280 °C, and held for 2 min. The MS was operated using an EI source with electron energy of 70 eV. Mass spectrometry used full scan mode with m/z ranging from 35 to 600. Linear retention indices (LRI) of the compounds were calculated using a linear alkane mixture (C7–C40) under the same conditions. After obtaining the ion flow diagram of volatile organic compounds, the qualitative analysis of the compounds was determined by matching MS with the NIST library, the retention time and retention index were compared with plant databases Pherobase (27 May 2021, www.pherobase.com), Flavornet (27 May 2021, www.flavornet.org/flavornet.html), NISTChemistry WebBook (27 May 2021, webbook.nist.gov/chemistry/), and literature reports [9,63,64].

### 4.3. Data Processing and Analysis

The relative content of VOCs was calculated by the peak area normalization method, which was expressed as the ratio of peak area to the total. Excel and R were used for all statistical analyses. Excel was used only for data sorting and statistics. Volatile values were presented as mean ± standard deviation (SD). The R version was 4.1.2. Analysis of variance (ANOVA) with Tukey’s test was used to assess volatile difference analysis. Different letters (a–c) in Tables S1 and S2 represent significant differences between samples (*p* ≤ 0.05). A Venn diagram was generated to visualize the shared and unique numbers of common and unique substances at different stages using R package “VennDiagram”. The alpha diversity index was calculated using R package “vegan”. Principal Component Analysis (PCA) was performed using the R packages “FactoMineR” and “factoextra”. All the visualization figures were implemented by R package “ggplot2”.

## 5. Conclusions

In this study, the VOCs profile of *R. willmottiae* in the wild was analyzed by SPME + GCMS. All the compounds can be divided into 10 categories, among which alcohols and terpenoids are most important. The flowering stages can be distinguished by the total content of substances. The contents of alcohols and esters are higher in the early and full flowering stages, whereas the contents of terpenoids are highest in the bud stages. Although the chemical diversity of double-flowers was not significantly different from that of single-flower, the cause of polyphyll type could be further explored in combination with other factors such as climate factors. Our result will provide a theoretical basis for the development and utilization of *Rosa willmottiae*. At the same time, it will open up new ideas for the economic development of Sichuan and Tibet.

## Figures and Tables

**Table 1:** α-diversity of VOCs (volatile organic compounds) of *Rosa willmottiae* at different flower phenotype.

Phenotype	Alpha Diversity Index		
	Shannon-Wiener	Simpson	Pielou
RW3 (single)	2.856	0.905	0.738
RWD (double)	2.896	0.912	0.740

## Supplementary Materials

The following are available online, Figure S1: *Rosa willmottiae* in the wild.; Table S1: The composition of VOCs (volatile organic compounds) in *Rosa willmottiae* at different stages and different flower phenotypes; Table S2: The richness and total content of VOCs classified by different categories in *Rosa willmottiae*.
