# Supplementary material for: Deeper Insight into the Volatile Profile of Rosa willmottiae with Headspace Solid-Phase Microextraction and GC–MS Analysis
Source: Molecules. 2022 Feb 12;27(4):1240. doi: 10.3390/molecules27041240 (PMC8874665; doi:10.3390/molecules27041240)
Supplement: Supplementary file 1 [file molecules-27-01240-s001.zip › molecules-1515225-supplementary.pdf]

Content：

Table S1. The composition of VOCs (volatile organic compounds) in *Rosa willmottiae* at different stages and different flower phenotype (RW1:bud stage RW2: initial flowering stage RW3: full flowering stage of single-flower phenotype RWD: full opening stage of double-flower phenotype, RT: Retention time)

Table S2. The richness and total content of VOCs classified by different categories in *Rosa willmottiae* (RW1: bud stage, RW2: early opening stage, RW3: full opening stage of single-flower phenotype, RWD: full opening stage of double-flower phenotype).

Figure S1. *Rosa willmottiae* in the wild. A. *Rosa willmottiae* in songpan B. single-flower phenotype C. double-flower phenotype

Table S1 The composition of VOCs in *Rosa willmottiae* at different stages and different phenotype (RW1:bud stage RW2: initial flowering stage RW3: full flowering stage of single-flower phenotype RWD: full opening stage of double-flower phenotype, RI: Retention Indices)

				Relative content （%）			
NO.	Chemicals	RI	CAS	RW1	RW2	RW3	RWD
1	2-ethylfuran	742	3208-16-0	0.33a±0.05	0.00b	0.00b	0.00b
2	2,3-demethyl-3-hexene	755	7145-23-5	0.00a	0.07b±0.01	0.04c±0.01	0.00a
3	hexanal	770	66-25-1	3.34a±0.59	0.94b±0.23	0.00c	0.00c
4	2-methyl-4-pentenal	798	5187-71-3	0.00a	0.00a	0.00a	0.61b±0.31
5	(E)-2-hexenal	809	6728-26-3	4.12a±0.04	2.73ab±0.27	1.57b±0.35	3.33a±0.92
6	2-hexenal	814	505-57-7	0.00a	0.00a	0.00a	0.11b±0.03
7	hexyl formate	858	629-33-4	0.46a±0.10	0.23ab±0.06	0.07b±0.02	0.42a±0.15
8	1,2-dimethyl-benzene	876	95-47-6	0.72bc±0.11	1.05a±0.02	0.92ab±0.13	0.56c±0.03
9	4-methyl-2-pentyl acetate	885	108-84-9	0.00a	0.04b±0.00	0.05b±0.01	0.00a
10	alpha-thujene	902	2867/5/2	0.10ab±0.02	0.05c±0.02	0.08bc±0.02	0.13a±0.01
11	benzaldehyde	925	100-52-7	7.50ab±0.64	6.08bc±0.28	5.62c±0.52	8.51a±0.56
12	(1R)- (+)-α-pinene	932	7785-70-8	0.72b±0.08	0.43c±0.05	0.58bc±0.08	1.03a±0.09
13	(1S)- (1)-β-pinene	978	18172-67-3	0.09a±0.02	0.00b	0.00b	0.14c±0.01
14	methoxymethyl benzene	984	538-86-3	0.10ab±0.02	0.13a±0.01	0.12ab±0.02	0.08b±0.01
15	6,6-dimethyl-2-methylene bicyclo [3.1.1] heptane	985	127-91-3	0.4a±0.02	1.42b±0.03	1.72b±0.11	1.31b±0.25
16	1,3,5-trimethylbenzene	1008	108-67-8	0.00a	0.29b±0.04	0.23b±0.03	0.00a
17	hexyl acetate	1008	142-92-7	0.00a	0.00a	0.00a	0.07b±0.01
18	m-cymene	1010	535-77-3	0.00a	0.00a	0.00a	0.03b±0.00
19	(R)-1-methyl-5-(1-methylvinyl) cyclohexene	1026	1461-27-4	0.09a±0.02	0.22b±0.03	0.29b±0.03	0.23b±0.03
20	benzyl alcohol	1032	100-51-6	10.02a±0.41	6.85b±0.77	10.69a±0.83	10.46a±0.37
21	phenylacetaldehyde	1032	122-78-1	0.51a±0.14	0.41a±0.11	0.28a±0.10	0.59a±0.09
22	3,7-dimethyl-1,3,6-octatriene	1041	13877-91-3	0.42a±0.02	0.63ab±0.09	0.69b±0.02	0.63ab±0.10
23	phenylethyl Alcohol	1043	60-12-8	14.48c±1.19	21.02a±1.06	20.49ab±0.83	18.08b±0.11
24	(E)-2-octenal	1057	2548-87-0	0.18a±0.03	0.00b	0.00b	0.00b
25	alpha-terpinolene	1084	586-62-9	0.00a	0.00a	0.00a	0.03b±0.00
26	nonanal	1084	124-19-6	0.14a±0.02	0.00b	0.00b	0.05c±0.00
27	linalool	1107	78-70-6	0.91a±0.18	0.31b±0.07	0.27b±0.06	0.28b±0.12
28	(E,Z)-2,6-dimethyl-2,4,6-octatriene	1129	7216-56-0	0.00a	0.18b±0.03	0.18b±0.04	0.00a

29	benzyl acetate	1163	140-11-4	0.49a±0.11	1.80a±1.00	1.73a±0.22	1.75a±0.31
30	alpha-Terpineol	1171	98-55-5	0.00a	0.00a	0.00a	0.13b±0.04
31	1,3-cyclohexadiene, 5-butyl-	1182	30168-57-1	0.11a±0.02	0.02b	0.00b	0.00b
32	2-methyl-4 undecane	1198	19594-40-2	0.11a±0.04	0.16a±0.01	0.14a±0.03	0.12a±0.00
33	dodecane	1199	112-40-3	0.00a	0.41b±0.08	0.16c±0.02	0.00a
34	nerol	1211	106-25-2	0.00a	0.21b±0.02	0.55c±0.13	0.00a
35	geraniol	1230	106-24-1	0.00a	0.77c±0.06	2.57b±0.75	2.11b±0.28
36	(Z,E)-3,7-dimethyl-2,6-octadienal	1244	5392-40-5	0.00c	0.57bc±0.05	0.98ab±0.20	1.23a±0.30
37	(E)-3,7-dimethyl-2,6octadienal	1244	141-27-5	0.00c	0.24b±0.03	0.38ab±0.08	0.51a±0.09
38	1,3,5-trimethoxybenzene	1248	621-23-8	0.75a±0.01	0.00b	0.00b	0.49c±0.09
39	acetic acid,2-phenylethyl ester	1265	103-45-7	0.67a±0.07	2.36b±0.52	3.58b±0.42	2.61b±0.47
40	2-methyl-1-indanone	1275	17496-14-9	0.17a±0.03	0.00b	0.00b	0.00b
41	(E, E)-2,4-decadienal	1281	25152-84-5	0.97a±0.23	0.69a±0.41	0.00b	0.00b
42	1h-inden-1-ol, 2,3-dihydro-2-methyl-	1296	17496-18-3	0.33a±0.06	0.00b	0.33a±0.06	0.00b
43	trans-2,4-decadienal	1312	2363-88-4	0.67a±0.15	0.00b	0.00b	0.00b
44	(+)-α-longipinene	1347	1493692	9.49a±0.54	6.19b±0.48	6.13b±0.22	11.25c±0.70
45	neryl acetate	1365	141-12-8	0.00a	0.70b±0.06	0.99c±0.06	0.11a±0.02
46	alpha- ylangene	1375	14912-44-8	0.47b±0.11	0.31bc±0.03	0.28c±0.04	0.69a±0.03
47	geranyl acetate	1383	105-87-3	0.00a	6.01b±0.35	8.47c±0.31	3.59d±0.11
48	tetradecane	1399	629-59-4	0.57a±0.06	0.00b	0.00b	0.00b
49	β-caryophyllene	1418	87-44-5	0.5ab±0.08	0.41b±0.04	0.60a±0.03	0.52ab±0.05
50	α-bergamotene	1431	17699-05-7	0.34a±0.08	0.23ab±0.04	0.26a±0.02	0.00b
51	β-maaliene	1432	489-29-2	11.43a±0.26	8.32b±0.75	8.66b±0.31	11.15a±0.62
52	gamma-muurolene	1477	30021-74-0	0.52a±0.08	0.39a±0.02	0.44a±0.01	0.90b±0.04
53	methyl isoeugenol	1491	93-16-3	2.45a±0.13	0.00b	0.00b	0.00b
54	δ-cadinene	1493	483-76-1	1.76a±0.08	1.39b±0.04	1.44b±0.04	2.34c±0.12
55	(-)-isocaryophyllene	1494	118-65-0	0.00a	0.53b±0.08	0.00a	a±0.00
56	pentadecane	1500	629-62-9	6.96a±0.66	5.49a±1.38	1.99b±0.17	2.56b±0.14
57	alpha-gurjunene	1529	489-40-7	0.00a	0.28b±0.03	0.00a	0.46c±0.02
58	(-)-alpha-muurolene	1541	10208-80-7	0.00a	0.16b±0.01	0.16b±0.02	0.27c±0.00
59	germacrene B	1544	15423-57-1	0.00a	0.00a	0.00a	0.24b±0.01
60	1,4-cadinadiene	1546	16728-99-7	0.00a	0.00a	0.04b±0.01	0.10c±0.01
61	(+)-palustrol	1548	95975-84-1	0.60ab±0.08	0.56b±0.04	0.55b±0.03	0.74a±0.03
62	alpha-calacorene	1548	21391-99-1	0.11a±0.02	0.00b	0.06c±0.01	0.00b
63	(E)-beta-farnesene	1636	28973-97-9	6.36a±0.75	5.01b±0.05	4.95b±0.17	1.44c±0.10
64	t-cadinol	1640	5937/11/1	0.00a	0.11b±0.00	0.15b±0.02	0.25c±0.02
65	α-muurolene	1644	31983-22-9	0.26a±0.04	0.01b	0.00b	0.00b
66	alpha-caryophyllene	1663	6753-98-6	0.84a±0.05	0.61b±0.01	0.85a±0.04	0.46b±0.07
67	beta-humulene	1710	116-04-1	0.00a	0.00a	0.27b±0.06	0.68c±0.02
68	heptadecane	1711	629-78-7	1.43a±0.06	1.40a±0.41	0.96ab±0.06	0.69b±0.04

69	β-bisabolene	1714	495-61-4	0.78a±0.07	0.00b	0.00b	0.00b
70	farnesen	1722	502-61-4	0.00a	0.50b±0.07	1.04c±0.04	1.04c±0.11
71	pentadecan-1-ol	1768	629-76-5	0.68a±0.07	0.43b±0.14	0.00c	0.00c
72	(E)-9-octadecene	1803	7206-25-9	1.40a±0.03	0.93b±0.28	0.00c	0.00c
73	eicosane	2000	112-95-8	0.31a±0.01	0.27a±0.06	0.00b	0.00b
74	kaurene	2025	34424-57-2	0.14a±0.01	0.06b0.01	0.05b±0.01	0.05b±0.01

Table S2 The richness and total content of substances classified by different categories in *Rosa willmottiae* (RW1: bud stage, RW2: early opening stage, RW3: full opening stage of single-flower phenotype, RWD: full opening stage of double-flower phenotype).

Chemical Category	Number				Relative content（%）			
	RW1	RW2	RW3	RWD	RW1	RW2	RW3	RWD
Alcohols	4	8	7	7	26.70c ±1.59	30.27bc±1.83	35.28a±0.89	32.05ab±0.23
Aldehyde	8	7	5	8	17.43a±1.44	12.04ab±0.57	7.62b±1.43	14.94a±2.31
Aliphatic hydrocarbons	5	6	4	2	10.66a±0.78	8.57a±2.20	3.15b±0.21	3.25b±0.19
Aromatic hydrocarbon	1	1	2	2	0.72a±0.11	1.34b±0.04	1.15b±0.16	0.58a±0.04
Esters	3	6	6	7	1.63a±0.16	11.15b±1.84	14.86c±0.72	8.55b±0.93
Ether	3	1	1	2	3.3a±0.10	0.13b±0.01	0.12b±0.02	0.57c±0.10
Ketone	2	1	1	1	0.28a±0.10	0.16b±0.01	0.14b±0.03	0.12b±0.01
Furan	1	0	0	0	0.33a±0.05	0.00b	0.00b	0.00b
Phenol	1	0	1	0	0.33a±0.06	0.00b	0.00b	0.00b
Terpenoids	15	17	18	22	34.94a±2.14	27.32b±1.60	28.77b±0.73	35.08a±1.6

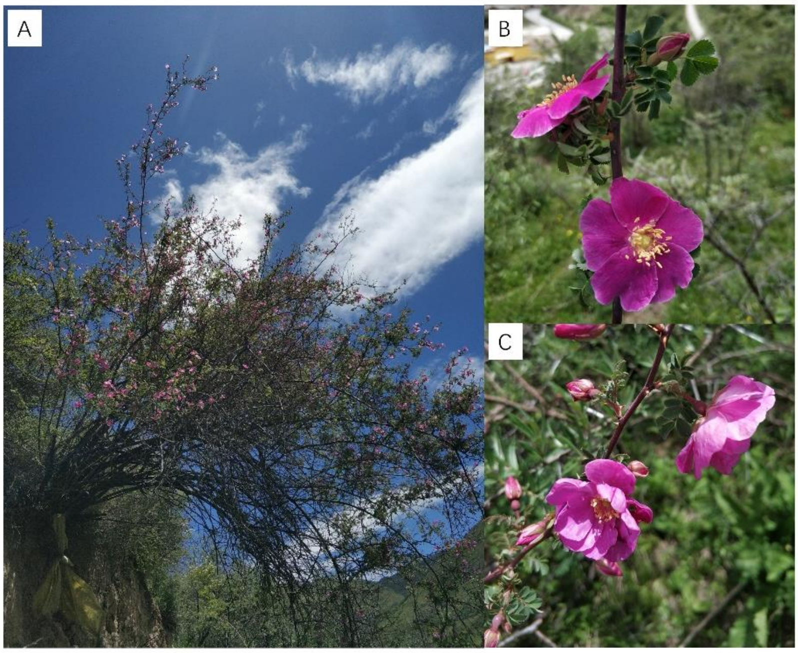

Figure S1. *Rosa willmottiae* in the wild. A. *Rosa willmottiae* in songpan B. single-flower phenotype C. double-flower phenotype
